# Spatiotemporal Transmission Dynamics of Hemorrhagic Fever with Renal Syndrome in China, 2005–2012

**DOI:** 10.1371/journal.pntd.0003344

**Published:** 2014-11-20

**Authors:** Wen-Yi Zhang, Li-Ya Wang, Yun-Xi Liu, Wen-Wu Yin, Wen-Biao Hu, Ricardo J. Soares. Magalhaes, Fan Ding, Hai-Long Sun, Hang Zhou, Shen-Long Li, Ubydul Haque, Shi-Lu Tong, Gregory E. Glass, Peng Bi, Archie C. A. Clements, Qi-Yong Liu, Cheng-Yi Li

**Affiliations:** 1 Institute of Disease Control and Prevention, Academy of Military Medical Science, Beijing, People's Republic of China; 2 Department of Infection Management and Disease Control, Chinese PLA General Hospital, Beijing, People's Republic of China; 3 Chinese Center for Disease Control and Prevention, Beijing, People's Republic of China; 4 School of Public Health and Social Work, Queensland University of Technology, Brisbane, Australia; 5 School of Veterinary Science, The University of Queensland, Brisbane, Australia; 6 WHO Collaborating Centre for Children Environmental Health, Queensland Children's Medical Research Institute, University of Queensland, Brisbane, Australia; 7 Emerging Pathogens Institute, University of Florida, Gainesville, Florida, United States of America; 8 Department of Geography, University of Florida, Gainesville, Florida, United States of America; 9 Discipline of Public Health, University of Adelaide, Adelaide, Australia; 10 Research School of Population Health, The Australian National University, Canberra, Australian Capital Territory, Australia; 11 State Key Laboratory for Infectious Disease Prevention and Control, National Institute for Communicable Disease Control and Prevention, Chinese Center for Disease Control and Prevention, Beijing, People's Republic of China; Tulane School of Public Health and Tropical Medicine, United States of America

## Abstract

**Background:**

Hemorrhagic fever with renal syndrome (HFRS) is a rodent-borne disease caused by many serotypes of hantaviruses. In China, HFRS has been recognized as a severe public health problem with 90% of the total reported cases in the world. This study describes the spatiotemporal dynamics of HFRS cases in China and identifies the regions, time, and populations at highest risk, which could help the planning and implementation of key preventative measures.

**Methods:**

Data on all reported HFRS cases at the county level from January 2005 to December 2012 were collected from Chinese Center for Disease Control and Prevention. Geographic Information System-based spatiotemporal analyses including Local Indicators of Spatial Association and Kulldorff's space-time scan statistic were performed to detect local high-risk space-time clusters of HFRS in China. In addition, cases from high-risk and low-risk counties were compared to identify significant demographic differences.

**Results:**

A total of 100,868 cases were reported during 2005–2012 in mainland China. There were significant variations in the spatiotemporal dynamics of HFRS. HFRS cases occurred most frequently in June, November, and December. There was a significant positive spatial autocorrelation of HFRS incidence during the study periods, with Moran's *I* values ranging from 0.46 to 0.56 (*P*<0.05). Several distinct HFRS cluster areas were identified, mainly concentrated in northeastern, central, and eastern of China. Compared with cases from low-risk areas, a higher proportion of cases were younger, non-farmer, and floating residents in high-risk counties.

**Conclusions:**

This study identified significant space-time clusters of HFRS in China during 2005–2012 indicating that preventative strategies for HFRS should be particularly focused on the northeastern, central, and eastern of China to achieve the most cost-effective outcomes.

## Author Summary

Hemorrhagic fever with renal syndrome (HFRS) is a rodent-borne viral disease caused by many serotypes of hantaviruses. In China, HFRS has been recognized as a severe public health problem and accounts for 90% of the reported cases in the world. We examined the spatiotemporal dynamics of HFRS cases in China during 2005–2012 and compared characteristics between cases from high-risk and low-risk counties. Several distinct HFRS cluster areas were identified, concentrated in northeastern, central, and eastern of China. Compared with cases from low-risk areas, a higher proportion of cases were younger, non-farmer, and floating residents in high-risk counties. These findings suggest preventative strategies for HFRS should be focused on the identified clusters in order to achieve the most cost-effective outcomes.

## Introduction

Hemorrhagic fever with renal syndrome (HFRS) is a viral zoonosis caused by different species of hantaviruses. The disease is characterized by fever, hemorrhage, headache, back pain, abdominal pain, acute renal dysfunction and hypotension [1]. In China, HFRS is mainly caused by two types of hantaviruses: *Hantaan* virus (HTNV) and *Seoul* virus (SEOV), which have *Apodemus agrarius* (striped field mouse) and *Rattus norvegicus* (brown rat), respectively as their major rodent hosts [2], [3]. Transmission of hantaviruses from rodents to humans is believed to occur through inhalation of aerosols contaminated by virus shed in excreta, saliva and urine of infected animals [4], [5].

HFRS was first recognized in northeastern China in 1931 and since 1955 it has spread to many other parts of China [6]. At present, it is widespread throughout the country and is endemic in all 31 provinces, autonomous regions, and metropolitan areas of China. HFRS has been recognized as a severe public health problem in China where it accounts for 90% of all reported cases in the world [3], [7], [8]. Currently, comprehensive control activities against HFRS including preventive measures (health education, rodent elimination two weeks before epidemic peak periods, vaccination to high-risk population and enhanced personnel protection), and control measures in epidemic periods (clearing and disinfection exposure environment, rodent control around the house, and reducing the contact with rodent in the workplace) have played important roles in the control of HFRS. However, HFRS still remains a serious public health problem with 20,000–50,000 cases reported annually [3], [7], [8]. With rapid economic development, urbanization and human population migration, together with the effects of climate change, new foci of infection have continuously emerged in recent years, and the endemic areas have extended from rural to urban areas and even into city centers [9]–[12]. The distribution of HFRS has varied geographically, and changed year by year. Previous studies have identified many regions in different provinces of China where clusters of HFRS have occurred [6], [10], [13]–[19].

In the past decades, spatiotemporal analysis techniques have been widely used in infectious disease surveillance and outbreak investigation [14], [20]–[28]. Spatiotemporal analyses help visualize epidemiological data, and detect and evaluate hotspots or clusters. Results may improve disease surveillance and efficiently manage control program resources [14], [20]–[28]. However, to the best of our knowledge, there has been no specific study on the spatiotemporal patterns of HFRS across China. A better understanding of the spatiotemporal distribution of HFRS would help to identify the areas, time and populations at highest risk, which would support the implementation of relevant and effective intervention measures.

In this study, we conducted geographic information system (GIS)-based analyses to characterize the spatiotemporal patterns of HFRS in mainland China using surveillance data from 2005–2012, to identify spatiotemporal clusters of HFRS cases at the county level, and to compare the demographic characteristics of HFRS cases from high-risk counties and low-risk counties as identified by the cluster detection analysis. The results from this study suggest strategies for regional and national HFRS control programs and enhanced intervention planning.

## Materials and Methods

### Ethics statement

The study was approved by the Ethics Committee of Beijing Institute of Disease Control and Prevention. In this study, all the data analyzed were anonymized for the consideration of confidentiality.

### Data collection and management

In China, HFRS has been a Class B Notifiable Disease since 1950. HFRS case reporting is compulsory to the Chinese Center for Disease Control and Prevention through the China Information System for Diseases Control and Prevention (CISDCP). Notified HFRS cases include information about sex, age, occupation, residential address, and onset date of symptoms for each case. Data from January 2005 to December 2012 were collected from CISDCP. In this study, all HFRS cases were confirmed according to the diagnostic criteria for HFRS from the Ministry of Health of the People's Republic of China [1]. The case definition for HFRS was an individual who had traveled to an HFRS endemic area or who had contacted with rodent feces, saliva, and urine within 2 months before the onset of illness, with clinical manifestations such as fever, chills, hemorrhage, headache, back pain, abdominal pain, acute renal dysfunction, and hypotension. In addition, the person had to meet at least one laboratory criteria for diagnosis: a positive result for hantavirus-specific immunoglobulin M, or a 4-fold rise in titers of hantavirus-specific immunoglobulin G, or a positive result for hantavirus-specific ribonucleic acid by reverse transcription polymerase chain reaction in clinical specimens, or hantavirus isolated from clinical specimens [1], [29]. Population data for each county during 2005–2012 were obtained from National Bureau of Statistics of China.

For the purpose of performing spatial analysis, the county was considered as the spatial unit for analysis. In mainland China, there are 2,922 counties, with population sizes ranging from 7,123 to 5,044,430 and area sizes from 5.4 to 197,346 square kilometers. All HFRS cases were geocoded and matched to the county-level polygons by administrative code, using the ArcGIS software (version 10.1, ESRI, Redlands, CA).

### Spatial smoothing using empirical Bayesian analysis

Spatial smoothing was used to reduce random variation associated with small populations and help identify spatial disease clusters that may not be apparent from direct observation of the raw data [23], [30]. We spatially smoothed the annual incidence rate over the 8-year period using an empirical Bayes spatial smoothing procedure found in GeoDa software (version 0.9.5-i). The smoothed incidence was calculated from the total number of cases in a county divided by the total number of people at risk within the county, which was specified using a spatial weights file generated by K-Nearest Neighbors algorithm [30]. Using this approach, a county with a small population at risk tended to have its observed rates adjusted considerably, whereas for larger counties the raw rates barely changed [30].

### Spatiotemporal cluster analysis

The analysis was conducted in three phases: First, assessing whether there was spatial autocorrelation in the annual incidence rate during 2 the study period using a global test for spatial autocorrelation (Moran's *I* index) using GeoDa software. A negative value of Moran's *I* indicates an overdispersed distribution, while a positive value indicates a clustered distribution, and a value around 0 indicates a spatially random distribution [31].

Second, to strengthen the confidence in the cluster analysis results, we conducted Local Indicators of Spatial Association (LISA) and Kulldorff's space-time scan statistic to explore the spatiotemporal clustering of HFRS. LISA was used to identify significant hotspots (High-High), coldspots (Low-Low), and outliers (High-Low and Low-High) by calculating local Moran's *I* index between a given location and the average of neighboring values in the surrounding locations [23], [32]. The significance of clusters was measured by a Z score, based on the randomization null hypothesis computation. A high positive Z score indicated that the surroundings had spatial clusters (High-High: high-value spatial clusters or Low-Low: low-value spatial clusters) and a low negative Z score meant spatial outliers (High-Low: high values surrounded with low values or Low-High: Low values surrounded with high values) [28]. Separate LISA analysis was performed each year for the average incidence rate of HFRS at the county level during the study periods using ArcGIS software.

Finally, to detect the location of high risk space-time clusters we used Kulldorff's space-time scan statistic (SaTScan software, version 9.1.1) [33]. The space-time scan statistic is defined by a cylindrical window with a circular (or elliptic) geographic base and with height corresponding to time [33]. The base is defined exactly as for the purely spatial scan statistic, while the height reflects the time period of potential clusters [33]. In this study, elliptic scan windows were used to fit discrete Poisson models. The maximum spatial cluster size was set to 10% of the population at risk in the spatial window and a maximum temporal cluster size of 20% of the study period in the temporal window. Likelihood ratio tests were performed to determine the significance of identified clusters and P-values were obtained through Monte Carlo simulation. The null hypothesis of a spatiotemporally random distribution was rejected when the P-value was <0.05 [28], [33].

### Comparison of demographic characteristics of HFRS between cases from high-risk and low-risk counties

To compare the characteristics of HFRS between cases from the high-risk counties with significantly greater than expected numbers of HFRS cases and low-risk counties with significantly fewer than expected HFRS cases identified by Kulldorff's spatiotemporal scan statistic, demographic data collected for each HFRS case were analyzed using χ^2^ tests or the *Z*-test. All statistical analyses were performed with SAS 9.2 (SAS Institute Inc., Cary, NC).

## Results

### Descriptive analyses

**Figure 1:**
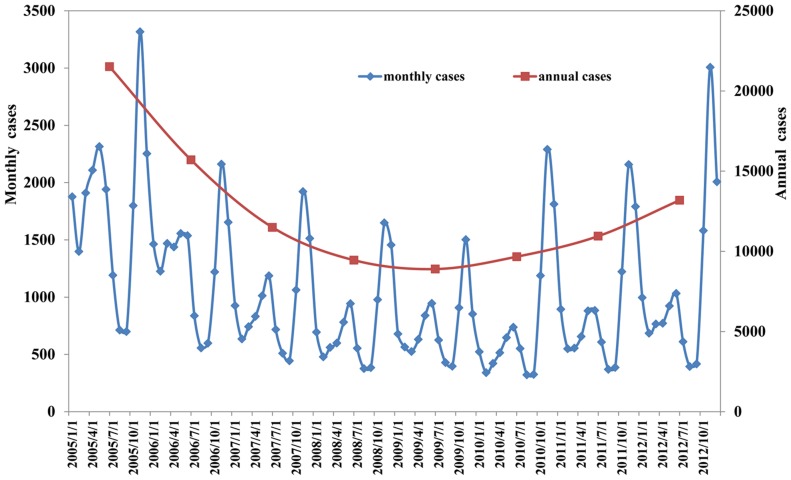
Temporal distribution of hemorrhagic fever with renal syndrome cases in mainland China.

**Figure 2:**
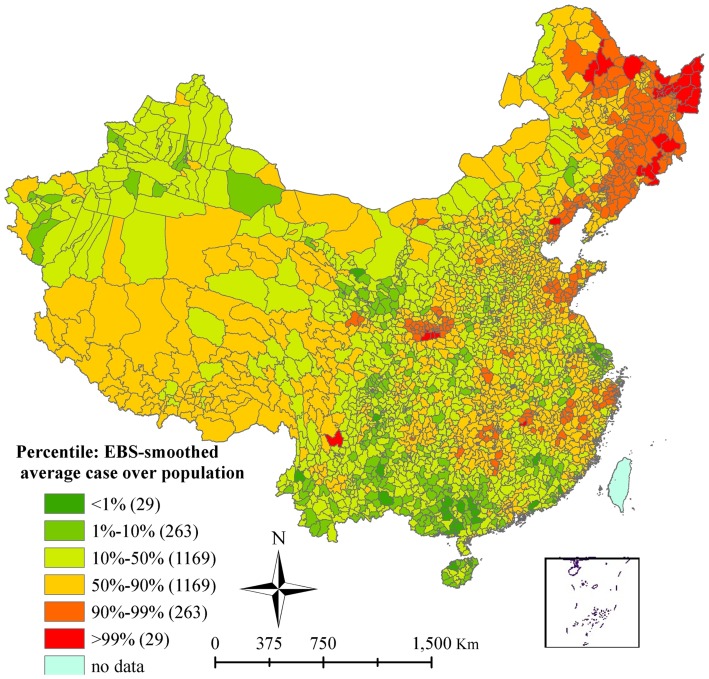
Spatial smoothed percentile map of hemorrhagic fever with renal syndrome using empirical Bayesian analysis, mainland China, 2005–2012.

A total of 100,868 HFRS cases were reported in 2,116 counties during 2005–2012 from a total of 2,922 counties. The monthly and annual variations of HFRS cases during the study period had a non-linear trend for HFRS cases in China, with bimodal seasonal distribution (Figure 1). HFRS decreased from 2005 to 2009 then increased after 2010. HFRS cases occurred throughout the year but had two seasonal peaks in June and November-December. These peaks accounted for 9.12% and 31.07% of cases respectively. The annual incidence rate of HFRS varied from 0 to 45.55/100,000 at the county level. Based on the smoothed estimates of incidence, HFRS varied geographically across the country, with northeastern China showing the highest overall risk (Figure 2).

### Spatiotemporal cluster analysis

There was significant positive spatial autocorrelation for HFRS incidence at the county level during the study period. Moran's *I* values ranged from 0.46 to 0.56 (Table 1), indicating that HFRS incidence rate was not randomly distributed, but was clustered.

**Table 1:** Spatial autocorrelation analysis for annual hemorrhagic fever with renal syndrome incidence in mainland China from 2005 to 2012.

Year	Moran's *I*	P-value
2005	0.5058	<0.001
2006	0.5161	<0.001
2007	0.5637	<0.001
2008	0.5089	<0.001
2009	0.4775	<0.001
2010	0.4602	<0.001
2011	0.5289	<0.001
2012	0.5581	<0.001

**Figure 3:**
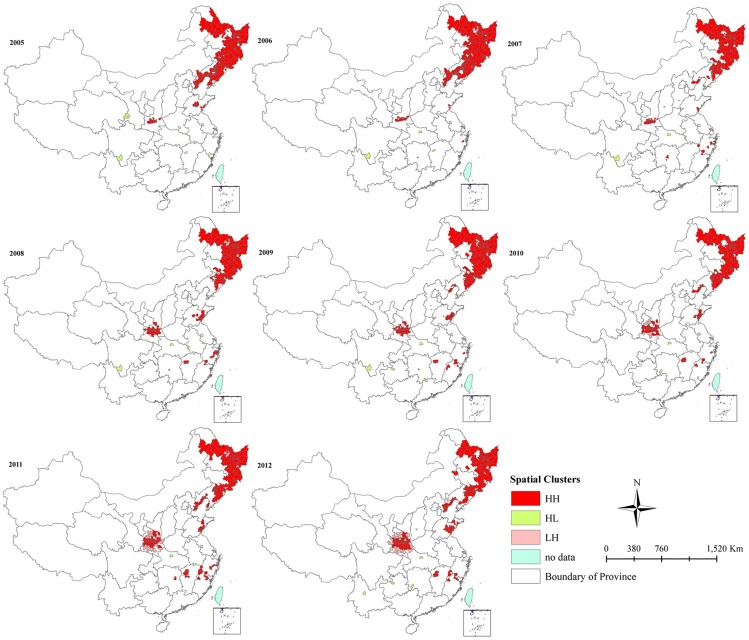
Yearly Local Indicators of Spatial Association (LISA) cluster maps for hemorrhagic fever with renal syndrome (HFRS) incidence, mainland China, 2005–2012. LISA spatial cluster map shows the center of the cluster in color. H-H indicates a statistically significant cluster of high HFRS incidence values; L-L indicates a cluster of low HFRS incidence values; L-H represents low HFRS incidence values surrounded with high HFRS incidence values.

LISA analysis identified hotspots (High-High) and outliers of HFRS transmission in mainland China (Figure 3). From 2005 to 2007, hotspots of HFRS transmission were mainly concentrated in northeast China and some parts of Shaanxi Province. However, a shift of hotspots was observed after 2008. The hotspots in Shaanxi and Shandong Province expanded and a sporadic appearance of hotspots in Jiangxi, Hunan, Fujian and Zhejiang Provinces, in southeast China, was observed from 2007 to 2012. Annual incidence rates in hotspots varied from 7.50/100,000 in 2009 to 17.95/100,000 in 2005 (Table 2). Hotspot counties comprised only a small percentage of total counties and total population of mainland China, ranging from 5.34% in 2007 to 7.73% in 2011 and 4.22% in 2007 to 6.32% in 2005, respectively, indicating the highly aggregated distribution of cases. A half, or more, of notified HFRS cases occurred in hotspots counties, ranging from 49.23% in 2007 to 62.84% in 2005.

**Table 2:** Descriptive statistics of hemorrhagic fever with renal syndrome spatial clusters as defined by a Local Indicators of Spatial Association analysis, mainland China, 2005–2012.

	Incidence rate* (1/100000)	% Cases	% Counties	% Population	% Area
2005					
HH	17.95	62.84	6.81	6.32	6.56
HL	21.44	1.67	0.21	0.12	0.22
LH	0.00	0.00	0.21	0.06	0.03
2006					
HH	12.30	56.94	6.98	6.10	6.63
HL	15.78	1.49	0.14	0.12	0.14
LH	0.00	0.00	0.24	0.04	0.07
2007					
HH	11.69	49.23	5.34	4.22	5.61
HL	10.54	1.92	0.21	0.16	0.15
LH	0.00	0.00	0.17	0.02	0.05
2008					
HH	8.07	56.42	6.47	5.59	5.97
HL	6.71	1.22	0.17	0.13	0.14
LH	0.00	0.00	0.27	0.03	0.09
2009					
HH	7.50	54.50	6.50	5.37	6.26
HL	5.01	1.48	0.24	0.19	0.17
LH	0.00	0.00	0.38	0.07	0.10
2010					
HH	7.90	55.01	6.13	4.94	5.84
HL	5.61	0.82	0.14	0.11	0.08
LH	0.00	0.00	0.48	0.19	0.24
2011					
HH	7.84	59.83	7.73	6.16	6.37
HL	7.25	0.33	0.03	0.04	0.02
LH	0.03	0.05	0.99	0.61	0.58
2012					
HH	9.92	61.56	7.53	6.27	5.95
HL	5.66	0.90	0.17	0.16	0.11
LH	0.06	0.05	0.99	0.78	0.69

Incidence rate*: annual incidence, calculated using yearly counts of HFRS cases as a numerator and population size in the middle year as a denominator; HH: High-High, a statistically significant cluster of high HFRS incidence values; LL: HL: High-Low, high HFRS incidence values surrounded with low HFRS incidence values; LH: Low-High, low HFRS incidence values surrounded with high HFRS incidence values.

**Figure 4:**
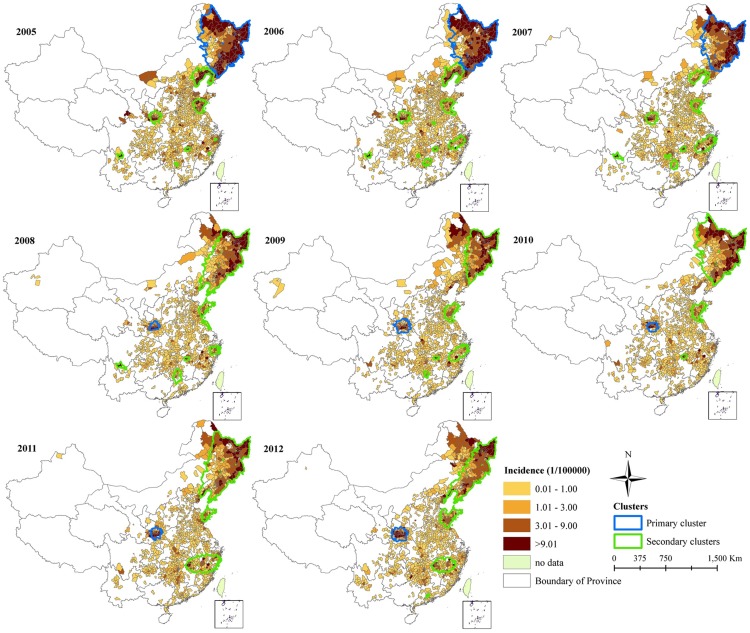
Distribution of yearly spatiotemporal clusters overlay with incidence of hemorrhagic fever with renal syndrome, mainland China, 2005–2012. Yearly spatiotemporal clusters were detected using an elliptic scan window with the maximum spatial size of 10% of the population at risk and a maximum temporal size of 20% of the study period.

Figure 4 shows a panel of maps of annual HFRS incidence and the location of spatial clusters identified using Kulldorff's spatial scan statistic, run independently for each year from 2005 to 2012 in mainland China. The primary cluster of HFRS cases occurred in Northeast China during 2005–2007. However, the primary cluster shifted to Shaanxi from 2008. Changes were also found in the secondary clusters of HFRS cases. There were 8, 9, and 8 secondary clusters during the years 2005, 2006, and 2007. Some clusters were not found in the subsequent years, with the number of secondary clusters ranging from 2 to 5.

**Figure 5:**
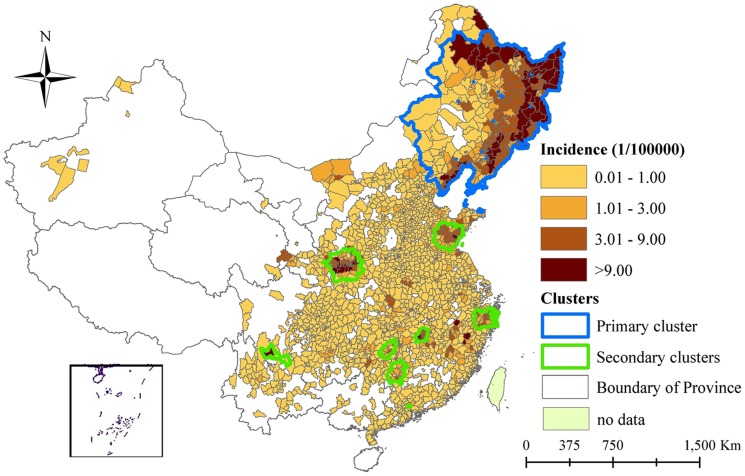
Spatiotemporal clusters overlay with annual average incidence of hemorrhagic fever with renal syndrome, mainland China, 2005–2012. Significant spatiotemporal clusters of hemorrhagic fever with renal syndrome from 2005 to 2012 were detected using an elliptic scan window with the maximum spatial size of 10% of the population at risk and a maximum temporal size of 20% of the study period.

Using Kulldorff's spatiotemporal scan statistic to detect space-time clusters across the entire study period (2005–2012), the primary cluster occurred in northeast China, including 126 counties in Heilongjiang, 104 counties in Liaoning, 64 counties in Jilin and 34 counties in Inner Mongolia (Figure 5). There were eight significant secondary clusters (cluster 2–cluster 9). Attributes of the clusters detected are shown in Table 3. The primary cluster was observed from January 2005 to July 2006, with a radius of 781 km, RR of 10.87, and LLR of 25,122. In addition, the primary cluster accounted for only 9.92% of the total population, but included 58.0% of the total number of cases during the cluster period (Table 4).

**Table 3:** Spatiotemporal clusters of hemorrhagic fever with renal syndrome in mainland China, during 2005–2012, detected using Kulldorff's spatiotemporal scan statistics*.

Clusters	E_Minor (Km)	E_Major (Km)	E_Angle (°C)	E_Shape	Time frame	No. Counties	No. Obs	No. Exp	LLR	RR
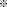 1	781	781	0	1.0	2005/1-2006/7	353	18001	1976	25121.92	10.87
#2	127	127	0	1.0	2011/10-2012/12	54	5189	271	10519.46	20.12
3	112	112	0	1.0	2005/1-2006/6	36	1980	376	1697.58	5.35
4	29	58	60	2.0	2010/11-2012/5	6	438	34	717.98	12.99
5	79	79	0	1.0	2005/12-2007/6	19	766	182	518.09	4.23
6	49	74	45	1.5	2006/11-2008/4	12	438	104	297.52	4.24
7	28	112	−30	4.0	2005/1-2006/6	4	219	17	357.57	12.88
8	67	67	0	1.0	2011/10-2012/12	12	241	81	103.71	3.00
9	12	12	0	1.0	2006/1-2007/4	3	96	38	30.43	2.50

* Significant clusters with P<0.01; 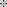
1: Primary cluster; #2-9: Secondary clusters; E_Minor: Semiminor axis of ellipse; E_Major: Semimajor axis of ellipse; E_Angle: the angle between the horizontal line and the semimajor axis of the ellipse; E_Shape: E_Major: E_Minor; No. Counties: number of counties within clusters; No. Obs: number of observed cases; No. Exp: number of expected cases; LLR: log likelihood ratio; RR: relative risk for the cluster compared with the rest of the country.

**Table 4:** Hemorrhagic fever with renal syndrome incidence rate, proportion of population and cases in spatiotemporal clusters in mainland China (2005–2012), detected using Kulldorff's spatiotemporal scan statistic*.

Cluster	Time frame	Incidence rate* (1/100,000)	% Population	% Case
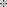 1	2005/1-2006/7	13.95	9.92	58.00
#2	2011/10-2012/12	23.26	1.72	28.27
3	2005/1-2006/6	7.63	2.00	6.56
4	2010/11-2012/5	19.85	0.17	2.15
5	2005/12-2007/6	6.44	0.91	3.29
6	2006/11-2008/4	6.14	0.55	2.48
7	2005/1-2006/6	18.64	0.09	0.73
8	2011/10-2012/12	3.64	0.51	1.31
9	2006/1-2007/4	3.22	0.23	0.51

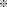
1: Primary cluster; #2-9: Secondary clusters; * Incidence rate: HFRS incidence during the clustering time; % Case: HFRS cases in cluster accounted for the total cases during the clustering time.

Table 5 shows the comparison of HFRS in cases from high-risk and low-risk counties as identified by Kulldorff's spatiotemporal scan statistic. Males accounted for a higher percentage of cases both in the high-risk counties and low-risk counties (76.85% vs. 75.48%). However, compared with cases from low-risk areas, a higher proportion of cases from high-risk areas were younger, non-farmer, and floating residents. HFRS cases from the high-risk counties had fewer days from illness onset to diagnosis with median days of 4 and 5 respectively (*P*<0.01) (Table 5).

**Table 5:** Comparison of characteristics of hemorrhagic fever with renal syndrome between cases from high-risk and low-risk counties identified by Kulldorff's spatiotemporal scan statistic in mainland China (2005–2012).

Variables	High-risk counties	Low-risk counties	Univariate analysis
**Sex**			
Male (%)	13833 (76.85)	62545 (75.48)	χ^2^ = 15.08, p<0.01
Female (%)	4168 (23.15)	20322 (24.52)	
**Age (year)**			
Median	39	43	Z = −34.44, p<0.01
Interquartile range	30–49	33–54	
**Occupation**			
Farmers (%)	12145 (67.47)	56368 (68.02)	χ^2^ = 2.08, p = 0.15
Non-farmers (%)	5856 (32.53)	26499 (31.98)	
**Address**			
Local Resident (%)	15445 (85.80)	72214 (87.14)	χ^2^ = 23.46, p<0.01
Floating Resident (%)	2556 (14.20)	10653 (12.86)	
**Days from illness onset to diagnosis**			
Median	4	5	Z = −26.74, p<0.01
Interquartile range	3.00–6.00	3–7.67	

## Discussion

There have been significant changes in the spatiotemporal dynamics of HFRS throughout mainland China during the recent past (2005–2012) including the appearance of a new, major cluster of HFRS in central China. This shift became evident by applying LISA and other spatial scan statistics analysis to historical surveillance data. Identifying clusters is of practical importance by providing health authorities with a rational basis to redirect their efforts to new or more specific high risk regions for environmental management, and implementing public health interventions, such as vaccinations and health education, in high-risk populations. Our study also demonstrates that GIS-based spatiotemporal analyses serve as useful tools to analyze the changing patterns of infectious diseases that have wider application in the field of surveillance and infectious disease management [6], [10], [13], [23], [24], [26], [28]. Additionally, these methods serve as an important strategy to better identify and characterize the dynamics of environmental correlates influencing pathogen maintenance.

Our study showed the remarkable variation in the spatiotemporal distribution of HFRS cases in China, with most high-risk counties located in the provinces of Heilongjiang, Jilin, Liaoning, Shaanxi and Shandong, where nearly half of all cases were located, indicating that HFRS still remains an important public health problem in China. Our findings helped policy-makers, public health practitioners and health authorities by identifying both the persisting, established focus in northeastern China and the reemerged newly identified foci which began in Shaanxi province in 2008 [9], [34], [35]. Our study also identified that the primary cluster of HFRS shifted to the center of Shaanxi province since 2008. Some studies have shown that the higher prevalence of infection and wider distribution of rodent hosts could drive the outbreak or reemergence of HFRS [9], [10]. The incidence of HFRS has been increasing in some big cities, such as Shenyang and Beijing [14], [15], [17]. Whether this reflects a shift in the primary rodent host responsible for most transmission or a change in population dynamics of the traditional species remains under investigation.

There are many environmental factors that impact the spatiotemporal dynamics of HFRS, such as changes in precipitation, temperature, land use, El Niño-Southern Oscillation, elevation, and vegetation community dynamics (as measured by Normalized Difference Vegetation Index) [3], [12], [29], [36]–[40]. The presumptive mechanisms for their actions on changing risk are varied. For example, temperatures may affect the dynamics and activities of rodent hosts as well as infectivity of hantavirus [41]. Variability in precipitation may influence the transmission of rodent-borne diseases by increasing the growth of vegetation, leading to larger rodent populations [42]. Our previous studies suggested that the climatic factors, therefore, might serve as leading indicators to predict changes in the risk of HFRS transmission in the province of Heilongjiang, Shandong and Beijing [13], [14], [40]. Socio-economic factors likely also play important roles in the transmission of HFRS [12], [14]. However, the cause for the expansion and increasing incidence of HFRS in some parts of mainland China are still unclear, and should be explored in future studies.

There are several limitations in our study. Firstly, the data are from a passive surveillance system in China, some asymptomatic cases (especially in low risk areas where clinical suspicion could be low) might be underreported. This might produce a bias in counts from these regions. However, quality control with data collection has been an important component of the disease-surveillance activities in China for all reportable diseases. For example, China CDC actively surveys hospitals and households to identify notifiable diseases each year. While we believe the quality assurance process minimizes the potential for spatial bias and under-reporting, the quality of data likely is not as good as the surveyed data. Secondly, relying on arbitrary aspects in some analytical methods may impact results. For example, the elliptic scan window was used in the space-time scan statistics. Although the elliptic scan window can identify the narrow, long, and noncontiguous areas better than the circular window option, this algorithm required more computational time [43]. Finally, in this study we didn't specifically incorporate the effects of rodent hosts, environmental characteristics, urbanization, sanitation and infrastructure, socio-economic data and control activities that drive the spatiotemporal patterns of HFRS incidence in China. Some of these data, such as detailed studies of rodent host dynamics throughout the entire country do not exist. Others, such as changes in the social structure of the country could be incorporated. Future studies will explore the role of socioeconomic impacting the spatiotemporal clusters identified in this study.

In conclusion, our results show that in the recent past HFRS cases were geographically concentrated in northeastern and central areas of China, but underlying this pattern is a shift in populations at risk for disease. This is the first study to examine the spatiotemporal dynamics of HFRS transmission in mainland China using selected spatial analytical methods and lays a foundation for further investigations into the social and environmental factors responsible for changing disease patterns. Our findings suggest that focusing preventative policies for HFRS in the high-risk counties enhance cost-effectiveness and health impact of HFRS control programs in China, while at the same time minimizing the environmental impacts that some interventions may have.
