# Correction: Spatiotemporal Transmission Dynamics of Hemorrhagic Fever with Renal Syndrome in China, 2005-2012

**DOI:** 10.1371/journal.pntd.0003599

**Published:** 2015-03-23

**Authors:** The PLOS Neglected Tropical Diseases Staff

The email address for one of the corresponding authors, Cheng-Yi Li, is listed incorrectly. The correct email address should be licy_60@163.com in the online version. The correct listing for corresponding author contact information in the PDF should be: *Email: licy_60@163.com (CYL); liuqiyong@icdc.cn (QYL).
